# Mechanical versus Bioprosthetic Aortic Valve Replacement in Middle-Aged Adults: A Systematic Review and Meta-Analysis

**DOI:** 10.3390/jcdd10020090

**Published:** 2023-02-20

**Authors:** Yefan Jiang, Song Wang, Jinhui Bian, Si Chen, Yongfeng Shao

**Affiliations:** 1 Department of Cardiovascular Surgery, The First Affiliated Hospital of Nanjing Medical University, Guangzhou Road, No. 300, Nanjing 210000, China; 2 Department of Cardiovascular Surgery, Union Hospital, Tongji Medical College, Huazhong University of Science and Technology, Jiefang Road, No. 1277, Wuhan 430022, China

**Keywords:** mechanical prostheses, bioprosthetic prostheses, clinical outcomes, middle-aged, meta-analysis

## Abstract

Background: Mechanical prostheses and bioprosthetic prostheses have their own advantages and disadvantages. Mechanical ones are recommended for younger patients (<50 years old), and bioprosthetic ones are recommended for older patients (>70 years old). There is still debate regarding which kind of prosthesis is better for middle-aged patients (50 to 70 years old) receiving aortic valve replacement (AVR). To solve this problem, we conducted this meta-analysis. Given that only one randomized controlled trial (RCT) study was included, we conducted a subgroup analysis of RCT and propensity score matching (PSM) retrospective studies to reduce the bias. Methods: We systematically searched articles related to clinical outcomes of mechanical and bioprosthetic prostheses in middle-aged patients receiving AVR in the PubMed, Cochrane Library, and China National Knowledge Infrastructure (CNKI) databases. The published date was up to 1 October 2022. Studies were excluded if not only middle-aged patients were included, or if they lacked direct comparisons between mechanical and bioprosthetic prostheses. Results: In total, 22 studies with 32,298 patients were included in the final analysis. The results show that patients aged between 50 and 70 receiving AVR with mechanical prostheses achieved better long-term survival and fewer reoperations and valve-related events but suffered more with bleeding events. No significant difference could be found in terms of early mortality and long-term cardiac death. The same results could be observed in the subgroup analysis of RCT and PSM retrospective studies. Conclusion: Both mechanical and bioprosthetic prostheses are beneficial to middle-aged patients undertaking AVR procedures. However, mechanical prostheses show better clinical outcomes in long-term survival and comorbidities. Individual recommendation is still necessary.

## 1. Introduction

Aortic valve disease is the most frequent heart valve disease in Western countries [1]. No effective medical treatments exist for this disease, and AVR remains the gold standard procedure [2]. AVR is indicated for survival benefit, symptom relief, and preservation of left ventricular function in patients with severe aortic valve disease [3]. Thus far, mechanical and bioprosthetic prostheses are clinically available for AVR procedures. It is acknowledged that mechanical prostheses are more likely to be recommended to younger patients (American Heart Association/American College of Cardiology guideline: <50 years old; European Society of Cardiology/European Association for Cardio-Thoracic Surgery guideline: <60 years old), whereas bioprosthetic prostheses are more likely to be recommended to elderly patients (American Heart Association/American College of Cardiology guideline: >70 years old; European Society of Cardiology/European Association for Cardio-Thoracic Surgery guideline: >65 years old) [4,5]. Although the use of bioprosthetic implantations has been increasing in all age periods in recent years, it is still undecided as to which prosthesis is better for middle-aged patients (aged from 50 to 70 years) [6,7]. Several studies have focused on this topic, but the conclusions are varied. Only one RCT study has been conducted, and it was by Stassano et al. [8]. Therefore, we conducted this meta-analysis to evaluate which kind of prosthesis is better for middle-aged patients receiving AVR: mechanical ones or bioprosthetic ones. Considering that only one RCT study was published, and PSM could eliminate a great proportion of systematic difference and permit a pooled analysis, the subgroup of RCT and PSM retrospective studies was also analyzed [9].

## 2. Methods

This meta-analysis was conducted following the Preferred Reporting Items for Systematic Review and Meta-Analysis (PRISMA) guidelines and has been registered with the International Prospective Register of Systematic Reviews (PROSPERO) (registration number: CRD42022365942).

### 2.1. Search Strategy

PubMed, Cochrane Library, and CNKI were searched for studies comparing clinical outcomes of mechanical and bioprosthetic prostheses in middle-aged patients receiving AVR up to 1 October 2022. We used “mechanical”, “bioprosthesis”, “bioprostheses”, “bioprosthetic”, “biological”, “aortic”, and “AVR” as key terms alone or in combination. The reference list of relevant articles and reviews was manually scrutinized to find additional studies. To check whether only patients aged from 50 to 70 years were included, we identified selected studies manually.

### 2.2. Eligibility Criteria

The inclusion criteria were as follows: (i) studies had to include a direct comparison of mechanical versus biological prostheses; (ii) clinical outcome information had to be provided in sufficient detail to allow the extraction of the hazard ratio (HR) or odd ratios (OR) and their standard errors or Kaplan–Meier curves. If several studies were produced at the same institution and there was a sample overlap, only the most updated study was included. Two authors (Dr. Yefan Jiang and Dr. Song Wang) independently extracted data from studies that met the inclusion criteria. Any differences were resolved by consensus or a discussion with a professional co-worker (Dr. Yongfeng Shao). Studies that met the inclusion criteria were rated based on the Newcastle Ottawa Scale (NOS), with three main components: study group selection, comparability between groups, and ascertainment of outcomes [10]. Paper quality was independently assessed by two authors (Dr. Yefan Jiang and Dr. Song Wang). A study with an NOS score of 6 or higher was regarded as being of high quality.

### 2.3. Statistical Analysis

The summary HR for long-term survival, freedom from cardiac death, freedom from reoperation, freedom from valve-related events, freedom from bleeding, and freedom from stroke and OR for early mortality were obtained as weighted averages of the measures from the individual studies, with inverse variances used as weights in the usual manner. The methods of Parmer et al., Williamson et al., and Tierney et al. were used to calculate the estimated HR and its variance [11]. A Q-statistic and an I2 (Index of Inconsistency) test were used to quantify the degree of heterogeneity in all studies. A random effect model was used in cases of significant heterogeneity (*p* < 0.1 or I2 > 50%). Sensitivity analyses were performed by omitting each study in sequence. Publication bias was assessed by the visual inspection of funnel plots. Data were analyzed with RevMan 5.3 (The Nordic Cochrane Center, The Cochrane Collaboration, Copenhagen, Denmark).

## 3. Results

### 3.1. Study Search

**Figure 1:**
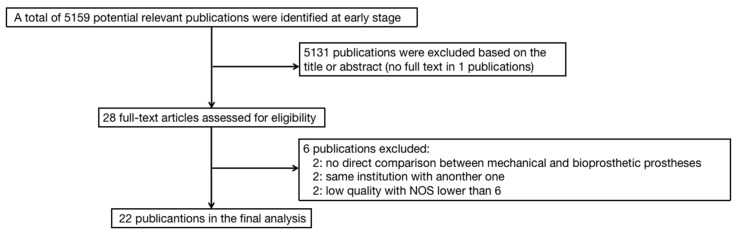
The flowchart outlining the literature search process.

The study search process is summarized in Figure 1. In total, 22 studies were included in the final analysis, and the characteristics of the individual studies are summarized in Table 1. Of those studies, one was an RCT study [8], 11 were PSM retrospective studies [1,3,12,13,14,15,16,17,18,19,20], and 10 were non-PSM retrospective studies [7,21,22,23,24,25,26,27,28,29]. In total, 32,298 patients were included, of which 15,449 received mechanical prostheses, whereas 16,849 received biological prostheses. All studies included had an NOS score of 6 or higher.

### 3.2. Early Mortality

**Figure 2:**
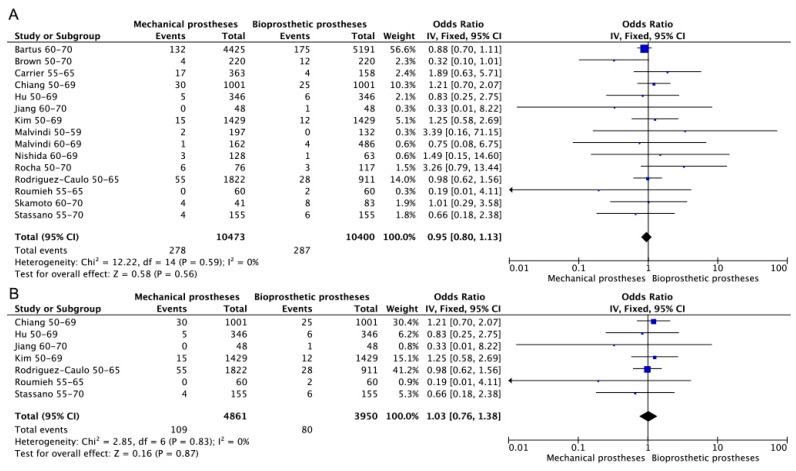
Meta-analysis for early mortality. (**A**) Bioprosthetic vs. mechanical. (**B**) Bioprosthetic vs. mechanical (RCT or PSM).

Early death refers to in-hospital death or death occurring within 30 days after operation. In total, 14 [3,7,8,13,15,16,19,20,21,22,23,24,26,27] studies provided related details, 7 [3,8,13,15,16,19,20] of which were RCT or PSM retrospective studies. No significant difference in early mortality existed between mechanical and bioprosthetic prostheses regardless of whether only RCT or PSM retrospective studies were included. (Bioprosthetic vs. mechanical—OR: 0.95; 95% CI: 0.80–1.13, Figure 2A. Bioprosthetic vs. mechanical (RCT or PSM)—OR: 1.03; 95% CI: 0.76–1.38, Figure 2B).

### 3.3. Long-Term Survival

**Figure 3:**
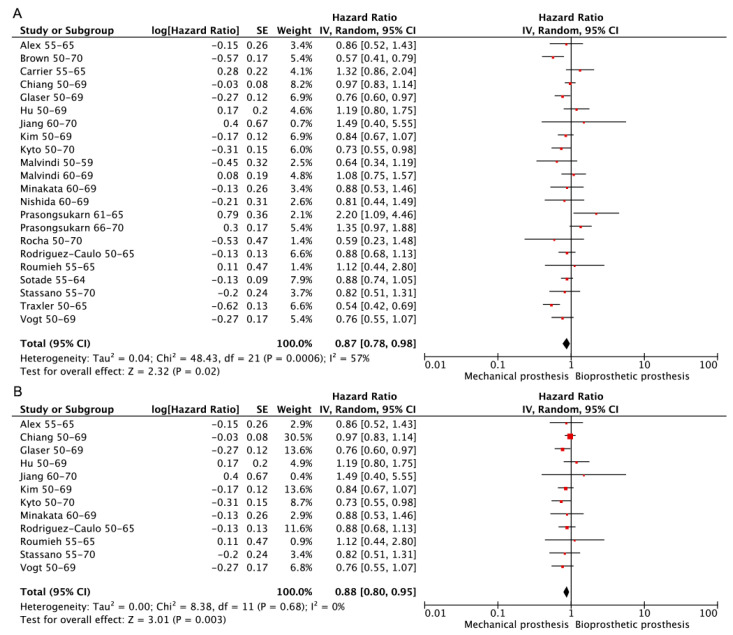
Meta-analysis for long-term survival. (**A**) Bioprosthetic vs. mechanical. (**B**) Bioprosthetic vs. mechanical (RCT or PSM).

Dates for long-term survival were available for 20 studies [1,3,8,12,13,14,15,16,17,18,19,20,21,22,23,24,25,26,28,29], 12 [1,3,8,12,13,14,15,16,17,18,19,20] of which were RCT or PSM retrospective studies. There was strong evidence favoring mechanical prostheses over bioprosthetic prostheses regardless of whether only RCT or PSM retrospective studies were included. (Bioprosthetic vs. mechanical—HR: 0.87; 95% CI: 0.78–0.98, Figure 3A. Bioprosthetic vs. mechanical (RCT or PSM)—HR: 0.88; 95% CI: 0.80–0.95, Figure 3B).

### 3.4. Freedom from Cardiac Death

**Figure 4:**
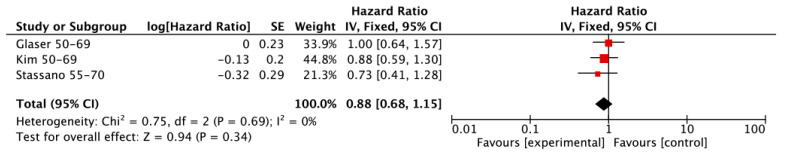
Meta-analysis for freedom from cardiac death: bioprosthetic vs. mechanical.

Only three studies [8,14,16] were included in this analysis, all of which were RCT or PSM retrospective studies. No difference in freedom from cardiac death was observed. (Bioprosthetic vs. mechanical—HR: 0.88; 95% CI: 0.68–1.15, Figure 4).

### 3.5. Freedom from Reoperation

**Figure 5:**
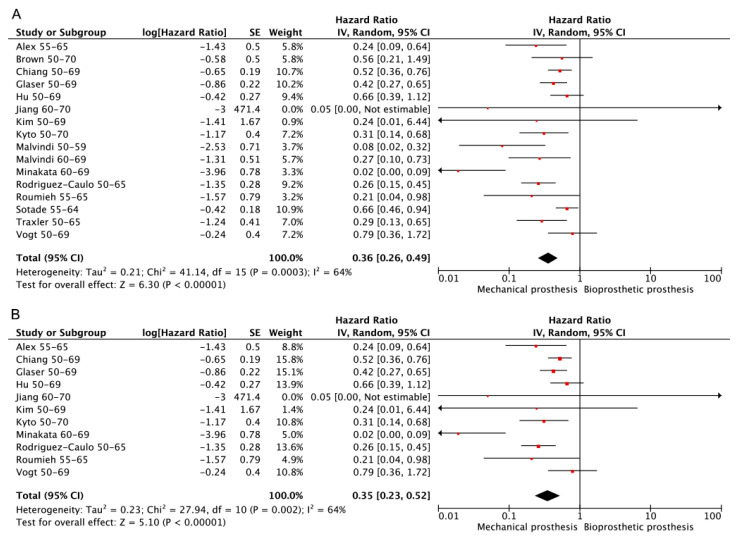
Meta-analysis for freedom from reoperation. (**A**) Bioprosthetic vs. mechanical. (**B**) Bioprosthetic vs. mechanical (RCT or PSM).

Of the 22 included studies, 15 [1,3,12,13,14,15,16,17,18,19,20,21,23,28,29] provided information that allowed the determination of freedom from reoperation, and 11 studies [1,3,12,13,14,15,16,17,18,19,20] were PSM retrospective studies. Significant heterogeneity existed in both analyses, and the random effect model was used. The exclusion of each study in sequence did not influence the overall results for both analyses. The results show that patients that received mechanical prostheses exhibited fewer reoperation events regardless of whether only RCT or PSM retrospective studies were included. (Bioprosthetic vs. mechanical—HR: 0.36; 95% CI: 0.26–0.49, Figure 5A. Bioprosthetic vs. mechanical (RCT or PSM)—HR: 0.35; 95% CI: 0.23–0.52, Figure 5B).

### 3.6. Freedom from Valve-Related Events

**Figure 6:**
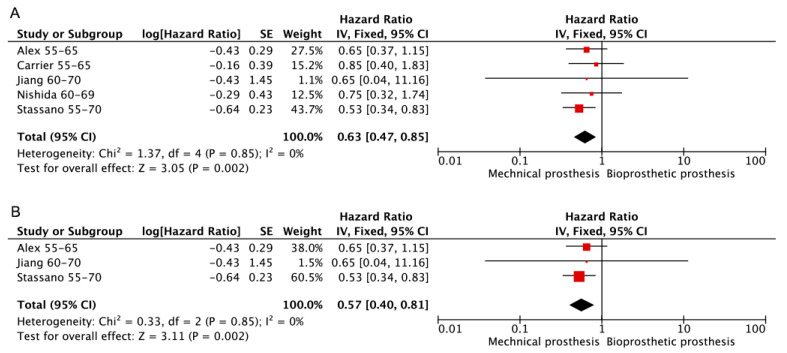
Meta-analysis for freedom from valve-related events. (**A**) Bioprosthetic vs. mechanical. (**B**) Bioprosthetic vs. mechanical (RCT or PSM).

All valve-related events are reported in accordance with the revised guidelines published by the “Ad. Hoc Liaison Committee for Standardizing Definitions for Prosthetic Heart Valve Morbidity (2008)” [11,30]. Five studies [8,12,15,22,24] provided sufficient details to allow the extraction of HR and their standard errors. Three [8,12,15] of these were RCT or PSM retrospective studies. Those analyses showed that the rate of valve-related events was lower in the mechanical prostheses group regardless of whether only RCT or PSM retrospective studies were included. (Bioprosthetic vs. mechanical—HR: 0.63; 95% CI: 0.47–0.85, Figure 6A. Bioprosthetic vs. mechanical (RCT or PSM)—HR: 0.57; 95% CI: 0.40–0.81, Figure 6B).

### 3.7. Freedom from Bleeding

**Figure 7:**
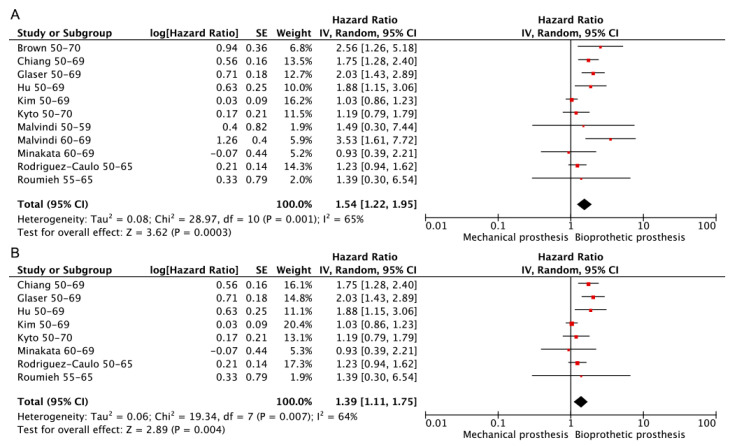
Meta-analysis for freedom from bleeding. (**A**) Bioprosthetic vs. mechanical. (**B**) Bioprosthetic vs. mechanical (RCT or PSM).

In total, 10 studies [3,13,14,16,17,18,19,20,21,23] reported information on freedom from bleeding, 8 [3,13,14,16,17,18,19,20] of which were PSM retrospective studies. The heterogeneity among those studies was relatively high, and the random effects model was used regardless of whether only PSM retrospective studies were included. The exclusion of each study in sequence did not influence the overall results. The results indicate that bleeding events were less common in bioprosthetic prostheses regardless of whether only RCT or PSM retrospective studies were included. (Bioprosthetic vs. mechanical—HR: 1.54; 95% CI: 1.22–1.95, Figure 7A. Bioprosthetic vs. mechanical (RCT or PSM)—HR: 1.39; 95% CI: 1.11–1.75, Figure 7B).

### 3.8. Freedom from Stroke

**Figure 8:**
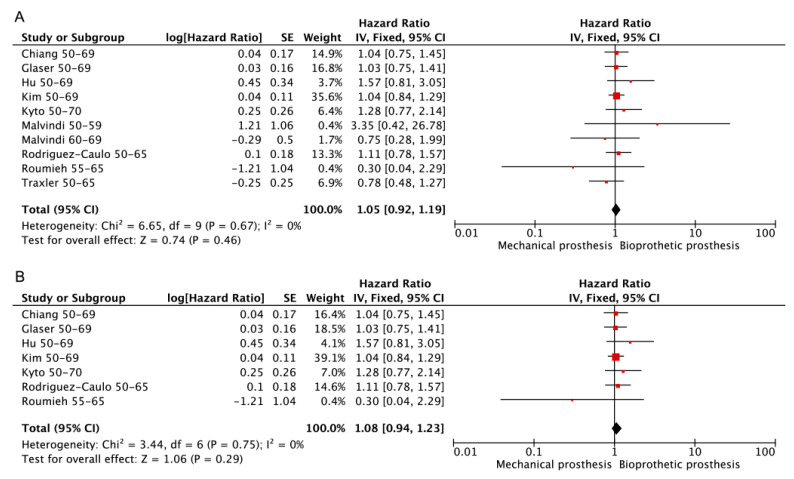
Meta-analysis for freedom from stroke. (**A**) Bioprosthetic vs. mechanical. (**B**) Bioprosthetic vs. mechanical (RCT or PSM).

Information on freedom from stroke was obtained in nine studies [3,13,14,16,17,19,20,23,29], seven [3,13,14,16,17,19,20] of which were PSM retrospective studies. No significant difference between mechanical prostheses and bioprosthetic prostheses could be observed regardless of whether only PSM retrospective studies were included. (Bioprosthetic vs. mechanical—HR: 1.05; 95% CI: 0.92–1.19, Figure 8A. Bioprosthetic vs. mechanical (RCT or PSM)—HR: 1.08; 95% CI: 0.94–1.23, Figure 8B).

## 4. Discussion

Mechanical and bioprosthetic prostheses have been widely used in patients with aortic valve disease. It is still undecided which kind of prosthesis is more suitable for middle-aged patients. No unequivocal recommendations are provided in the current published guidelines, and only ambiguous suggestions are given [4,5]. Several studies have focused on this topic, but the conclusions are inconsistent. Chiang et al. reported that no difference exists in the long-term survival between mechanical and bioprosthetic prostheses, but patients receiving bioprosthetic ones had a greater likelihood of reoperation and a lower likelihood of major bleeding [13]. Glaser et al. reported that patients aged 50–69 years who received mechanical prostheses had better long-term survival than those with bioprostheses. The risk of stroke was similar. However, patients with bioprostheses had a higher risk of reoperation and a lower risk of major bleeding [14]. Two systematic reviews on this topic have been published [9,31], but several limitations exist in those two meta-analyses. Firstly, only a few published articles were included. Secondly, the conclusions were not consistent. Zhao et al. stated that no significant difference existed in the long-term survival between mechanical and bioprosthetic prostheses in patients aged 50 to 70 years. Compared with mechanical prostheses, bioprosthetic ones showed reduced risk of major bleeding and anticoagulant-related events but increased risk of reoperation [31]. Diaz et al. showed that mechanical prostheses were associated with a long-term survival benefit, lower reoperation rates, higher bleeding rates, and similar stroke rates for patients between the ages of 50 and 70 [9]. The main contentions regard long-term survival and stroke rates, whether in meta-analyses or in normal retrospective studies. We also focus on those two aspects in our study. Thirdly, these two analyses were published in 2016 and 2018; several retrospective studies have been published since then, most of which were multi-center and even nationwide, which may provide more accurate results [3,16,23,28,29,32,33]. Therefore, we conducted this meta-analysis to further compare the clinical outcomes of mechanical and bioprosthetic prostheses in middle-aged patients receiving AVR surgery to give clinicians an update on this topic. Because only one RCT study was published related to this topic, we also performed a subgroup analysis of RCT and PSM retrospective studies to reduce bias accompanied with retrospective studies. 

The middle-age period is awkward. These individuals are older than those younger than 50 and have longer life expectancy than those older than 70. Choosing mechanical prostheses means life-long anticoagulation, regular International Normalized Ratio (INR) value monitoring, and a higher bleeding risk. Choosing bioprosthetic ones entails the possibility of reoperation. You cannot have your cake and eat it. Previous studies have suggested that the choice in prosthesis depends on expected lifespan. For patients with expected lifespans below 20 years, bioprosthetic prostheses were recommended, and for patients with expected lifespans over 20 years, mechanical ones were recommended [34]. However, it is hard to determine middle-aged patients’ expected lifespan exactly.

In our analysis, we found that in patients aged between 50 and 70 following AVR, the early mortality, cardiac death rates, and stroke rates were similar. However, patients receiving mechanical prostheses showed decreased long-term risk of death and fewer reoperation and valve-related events. The only superiority for bioprosthetic prostheses was fewer bleeding events. These results are independent of whether only RCT and PSM retrospective studies are considered. According to the latest American Heart Association/American College of Cardiology guideline (2020), bioprosthetic prostheses are recommended for patients older than 65, whereas mechanical prostheses are recommended for patients younger than 50 [35]. Only eight studies consider patients aged from 50 to 65. We performed a subgroup analysis to detect which kind of prosthesis is more suitable for patients aged from 50 to 65 years old (Supplementary Figures S1 and S2). The early mortality, long-term survival, reoperation, valve-related events, bleeding, and stroke rates were similar between the two groups. Patients receiving mechanical prostheses showed fewer reoperation events. These results are independent of whether only RCT or PSM retrospective studies are included.

These results can be explained mainly by considering the prosthetic materials. Mechanical prostheses are constructed of pyrolytic carbon, whereas bioprosthetic prostheses are made of bovine, equine, or porcine pericardium or porcine aortic valves. Due to the distinct characteristics of the materials used, mechanical and biological prostheses have their own advantages and disadvantages. Mechanical ones can be used for life, and fewer structural valve deterioration events occur. However, because of the thrombogenicity features of materials used in mechanical prostheses, patients require life-long anticoagulation therapy to avoid blood clot formation; thus, bleeding events are more frequent in patients receiving mechanical prostheses. Distinct from the advantages of mechanical prostheses, the advantage of bioprosthetic ones is that patients do not require life-long anticoagulation. However, similar to pathological processes, bioprosthetic implantation also entails cusp calcification, cusp tears, perforation, stretching, thickening, stiffening, and prolapse, all of which lead to more reoperations in patients with bioprosthetic prostheses [6,7,31,36].

Better long-term survival may not only be related to material features but may also be associated with the fact that prosthesis–patient mismatch may occur more often in patients with bioprosthetic prostheses, which might affect long-term survival [37]. With the same annular size, mechanical prostheses usually present a higher index of effective orifice area values than bioprosthetic prostheses, and the rate of postoperative prosthesis–patient mismatch could be lower. Thus far, the related information is limited, and fewer studies have focused on this aspect. Further investigation is needed.

Stroke is the result of thrombosis. Although life-long anticoagulation is not recommended for patients receiving bioprosthetic prostheses, thrombosis still exists. Recent studies have shown that multislice computed tomography during follow-up identified 7% of patients receiving bioprosthetic prostheses after AVR to have reduced leaflet motion as the result of thrombosis [38]. This may partly explain the similarity in stroke rates between the two groups. Some patients with bioprosthetic prostheses also receive warfarin or clopidogrel bisulfate at late follow-up due to their own comorbidities, such as atrial fibrillation, transient ischemic attacks, and stroke, all of which may increase the complexity of the comparison [21]. In addition, with the use of anticoagulation therapy, the stroke rates in patients with mechanical prostheses were decreased, which may also partly explain the similarity in stroke rates between the two groups. Finally, some AVR patients with microembolism were asymptomatic, and head scanning is necessary for the identification of those patients. The existence of those potential stroke patients may disturb the comparison.

With the development of technology, several improvements in favor of mechanical prostheses have appeared. Firstly, non-vitamin K antagonist anticoagulation may reduce the bleeding rates in patients receiving mechanical prostheses [39]. A pilot study proved that Rivaroxaban (20 mg once daily) was safe and effective in low-risk patients with mechanical prostheses. Neither thromboembolic nor bleeding events were observed, and no patients died [40]. Secondly, a lower INR range may be applied. The risk of bleeding can be significantly reduced if the targeted INR can be lowered [6]. Koertke et al. conducted a prospective, randomized multi-center trial. In total, 1346 patients with a target INR range of 2.5–4.5 and 1327 patients with a target INR range of 1.8–2.8 for AVR and an INR range of 2.5–3.5 for mitral or double-valve recipients were followed up for 24 months. The incidence rates of stroke and bleeding were similar between the two groups [41]. Of course, further research is necessary to clarify to what extent the INR target can be lowered. Thirdly, new mechanical designs have been developed. The On-X valve permits a lower INR range of 1.5–2.0 in the aortic position [42]. Besides this, Scotten et al. developed a 3D-printed mechanical prosthesis with a lower thrombogenicity potential index, which may function without the need for anticoagulation [43].

With the introduction and widespread application of transcatheter aortic valve implantation, valve-in-valve procedures have emerged as a valuable option in patients with degenerated bioprosthetic prostheses [44]. The prospect of implanting a bioprosthesis with a subsequent valve-in-valve may influence the decision to implant a mechanical or bioprosthetic prosthesis in middle-aged patients and may encourage middle-aged patients to choose bioprosthetic prostheses [6]. However, the mortality related to valve-in-valve procedures is still high. Phan et al. reported that for patients with degenerated bioprosthetic prostheses, the early mortality was 7.9% in patients receiving valve-in-valve procedures, whereas the early mortality was 6.1% in patients receiving conventional aortic valve replacement [45]. Besides this, valve-in-valve procedures were accompanied with decreased effective orifice areas due to the previously implanted prostheses, which may lead to postoperative prosthesis–patient mismatch, especially for patients with a small annulus [46]. Experience with valve-in-valve procedures remains relatively limited, and long-term follow-up is necessary.

### Limitations

Several limitations of this meta-analysis should be noted. Firstly, only one RCT study was included, and the other studies included were all retrospective studies, which may increase the bias, although a subgroup analysis of RCT and PSM studies was performed. Secondly, the operative years reported in the studies had a broader range, which could have reduced the comparability of the studies in the analysis. Thirdly, the size, the brands, and the accompanied surgeries were not identified in our study, which may increase the bias.

## 5. Conclusions

In conclusion, for middle-aged patients receiving AVR, both kinds of prostheses have their own advantages and disadvantages, but mechanical prostheses may be associated with better long-term survival and fewer reoperation and valve-related events. Of course, individual preference is much more important. To reach more accurate conclusions, more RCTs should be conducted in future.

## Figures and Tables

**Table 1:** The characteristics of the individual studies.

Study Name	Country	Study Period	Age Period	Number of Prostheses		Mean Age(years)		Male		Concomitant Operation		Follow-Up Duration(years)		Study Design
				MP	BP	MP	BP	MP	BP	MP	BP	MP	BP	
Alex [12]	Canada	1995–2014	55–65	118	118	62	61.5	79	84	CABG	CABG	15	15	PSM
Bartus [7]	Poland	2006–2016	60–70	4425	5191	63.9	65.7	2761	3058	-	-	-	-	Retrospective
Brown [21]	USA	1991–2000	50–70	220	220	65.7 ± 3.9	66.6 ± 4.1	156	154	CABG	CABG	9.1	6.2	Retrospective
Carrier [22]	Canada	1982–1999	55–65	363	158	61 ± 3	61 ± 3	254	120	CABG	CABG	4 ± 3	7 ± 5	Retrospective
Chiang [13]	USA	1997–2004	50–69	1001	1001	61.5 ± 5.3	61.5 ± 5.7	645	634	isolated		10.8		PSM
Glaser [14]	Sweden	1997–2013	50–69	1099	1099	62.3 ± 4.5	62.1 ± 5.1	719	747	isolated		-	-	PSM
Hu [3]	China	2002–2018	50–69	346	346	62.3 ± 5.5	62.7 ± 5.1	220	211	CABG		6.5		PSM
Jiang [15]	China	2005–2015	60–70	48	48	62.8 ± 2.2	63.1 ± 2.4	33	32	CABG		-	-	PSM
Kim [16]	Korea	2002–2018	50–69	1429	1429	62.9 ± 4.3	63.1 ± 4.2	900	907	isolated		5		PSM
Kytö [17]	Finland	2004–2014	50–70	576	576	64.6 ± 4	65.1 ± 4.5	399	393	CABG		6.7		PSM
Malvindi [23]	Poland	2000–2019	50–59	197	132	55 ± 3	56 ± 3	124	90	isolated		9.3	4.7	Retrospective
			60–69	162	486	64 ± 3	66 ± 3	103	289	isolated		10.7	5.4	
Minakata [18]	Japan	1985–2001	60–69	220	92	-	-	-	-	-	-	-	-	PSM
Nishida [24]	Japan	1981–2013	60–69	128	63	64.1 ± 0.2	65.6 ± 0.3	67	52	CABG, AAR, MVP, Maze procedure		9.8 ± 0.6	6.7 ± 0.7	Retrospective
Prasongsukarn [25]	Canada	1982–1998	61–65	150	153	63.4 ± 1.5	63.7 ± 1.4	103	134	CABG		6.4 ± 3.2	8.9 ± 5.3	Retrospective
			66–70	195	424	68.3 ± 1.3	68.7 ± 1.4	90	269	CABG		5.6 ± 2.9	8.2 ± 4.6	
Rocha [26]	Portugal	2012	50–70	76	117	59.5	63	40	72	MVI, TVI, multivalve, CABG, AAS		7		Retrospective
Rodríguez-Caulo [19]	Spain	2000–2018	50–65	1822	911	60.8 ± 3.9	60.9 ± 4.1	1229	610	-	-	8.5 ± 4.8	7.3 ± 4.8	PSM
Roumieh [20]	Germany	1996–2008	55–65	60	60	61 ± 3	61.5 ± 3	43	43	isolated		10.7 ± 4.5	8.8 ± 3.8	PSM
Skamoto [27]	Japan	1995–2014	60–70	28	28	64.3 ± 2.8	65.4 ± 2.6	16	12	CABG		7.0 ± 5.6	7.8 ± 5.7	Retrospective
Sotade [28]	Australia	2003–2018	55–64	1319	1522	61	61	975	1104	CABG		7	6	Retrospective
Stassano [8]	Italy	1995–2003	55–70	155	155	64 ± 7.6	63.5 ± 3.9	66	78	CABG		8.3 ± 2.3		RCT
Traxler [29]	Australia	2010–2018	50–65	702	1910	60		1883		-	-	-	-	Retrospective
Vogt [1]	Germany	2011–2012	50–69	610	610	58.2 ± 4.5	58.2 ± 4.5	431	437	isolated		-	-	PSM

AAR: ascending aorta replacement; AAS: ascending aorta surgery; BP: bioprosthetic prostheses; CABG: coronary artery bypass grafting; MP: mechanical prostheses; MVP: mitral valve plasty; MVI: mitral valve intervention; PSM: propensity score matching; RCT: randomized controlled trial; TVI: tricuspid valve intervention.

## Data Availability

The data presented in this study are available on request from the corresponding author.

## Supplementary Materials

The following supporting information can be downloaded at: https://www.mdpi.com/article/10.3390/jcdd10020090/s1. Figure S1: Subgroup analysis of patients aged from 50 to 65 years old. (A) Early mortality. (B) Long-term survival. (C) Freedom from reoperation. (D) Freedom from valve-related events. (E) Freedom from bleeding. (F) Freedom from stroke. Figure S2: Subgroup analysis of patients aged from 50 to 65-years-old (RCT or PSM). (A) Early mortality. (B) Long-term survival. (C) Freedom from reoperation. (D) Freedom from valve-related events. (E) Freedom from bleeding. (F) Freedom from stroke.
