# Supplementary figures and images for: Mechanical versus Bioprosthetic Aortic Valve Replacement in Middle-Aged Adults: A Systematic Review and Meta-Analysis
Source: J Cardiovasc Dev Dis. 2023 Feb 20;10(2):90. doi: 10.3390/jcdd10020090 (PMC9965629; doi:10.3390/jcdd10020090)

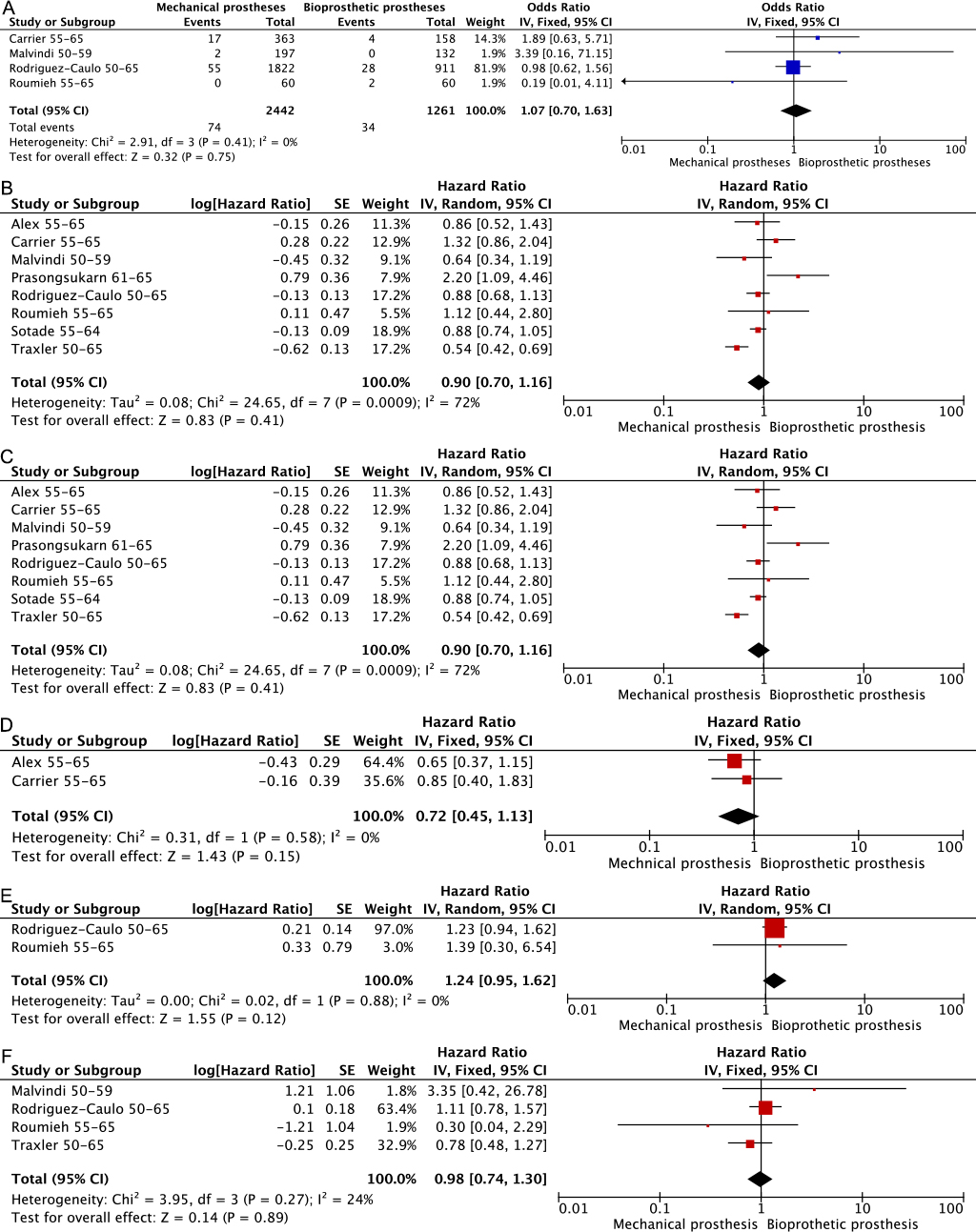

Supplement: Supplementary file 1 [file jcdd-10-00090-s001.zip › supplementary figure S1.jpg]

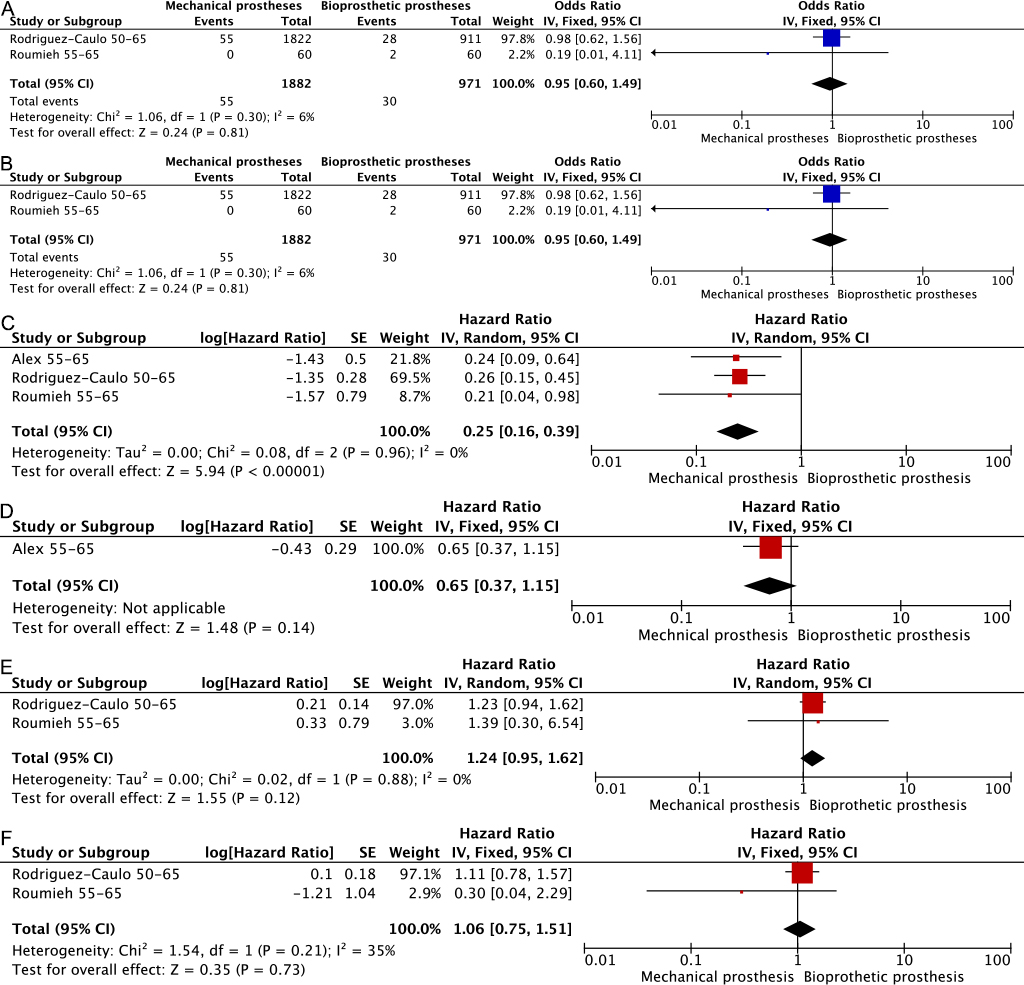

Supplement: Supplementary file 1 [file jcdd-10-00090-s001.zip › supplementary figure S2.jpg]
